# Ecological Momentary Assessment of the Quality of Life and Self-Efficacy Among People With a Stoma: Longitudinal Study

**DOI:** 10.2196/57427

**Published:** 2025-07-02

**Authors:** William Goodman, Matthew Allsop, Amy Downing, Julie Munro, Gill Hubbard, Rebecca J Beeken

**Affiliations:** 1 School of Medicine, University of Leeds, Worsley Building, Leeds, LS2 9JT, United Kingdom, 44 0113 343 0741; 2 NHS Highland, Inverness, United Kingdom; 3 School of Health Sciences, University of Dundee, Dundee, United Kingdom

**Keywords:** ecological momentary assessment, quality of life, self-efficacy, stoma, digital health, abdomen, ileostomy, bowel cancer, bowel disease, opening, colostomy, stigma, daily routine, smartphone, mHealth, mobile application, app, clinical, multilevel modelling, self-management, intervention, inflammatory bowel disease

## Abstract

**Background:**

When a stoma is formed, people with a stoma have to adjust to managing this on a daily basis. There are a number of factors that can impact their self-efficacy to self-manage and their quality of life, including complications with their stoma, body image concerns, stigma, and the changes to their daily routine. Ecological momentary assessment studies in other populations have suggested that these constructs may vary over short periods of time and could be influenced by contextual factors. We, however, do not currently understand how context-specific factors such as what an individual is doing, who they are with, or where they are could impact upon quality of life and self-efficacy in people with a stoma.

**Objective:**

This study aimed to understand whether quality of life and self-efficacy fluctuated over time and whether contextual factors (eg, activity, location, or company) and time of day were associated with quality of life and self-efficacy over the course of a week.

**Methods:**

A smartphone-based ecological momentary assessment study was conducted over a 7-day period with measurements taken 3 times a day (morning, afternoon, and evening). Measures included demographic and clinical characteristics of age, gender, and time with a stoma, and self-reported self-efficacy and quality of life in the moment rated from 0 to 100 (with 100 being the best). Multilevel modeling was conducted due to the clustering of responses within individuals, with models run for both self-efficacy and quality of life.

**Results:**

In total, 62 participants completed the ecological momentary assessment protocol. Null multilevel models indicated that both self-efficacy and the quality of life varied over time, with around 35% of total variance explained by within-person variability, which suggests that there are intraindividual fluctuations over time. Results indicated that, for the self-efficacy model, people reporting from home had higher levels of self-efficacy than those outside the home (*β*=−3.8, 95% CI −6.4 to −1.3). In the quality of life model, there was increasing levels of quality of life throughout the day (afternoon: *β*=2.0, 95% CI 0.8-3.2, evening: *β*=3.9, 95% CI 2.6-5.2).

**Conclusions:**

To the best of our knowledge, this is the first study to use ecological momentary assessment in this population. These findings indicate that, among people with a stoma, self-efficacy and quality of life do vary from moment to moment. Furthermore, contextual factors such as location and time of day are associated with self-efficacy and quality of life. There is a need to explore how future self-management interventions could enhance self-efficacy to self-manage outside of the home environment, with the potential for more dynamic and tailored interventions.

## Introduction

A stoma is an artificial opening on the abdomen created to divert the flow of feces or urine; this paper focuses on the two bowel stomas, ileostomies and colostomies [1]. There are no global estimates for the number of people with a stoma, although estimates for the United States and China suggest there are around 1 million people with a stoma in each, with 700,000 in the European Union [2,3]. A stoma is primarily created due to bowel cancer but can also be formed due to inflammatory bowel disease (IBD), physical trauma, and other reasons (eg Hirschsprung’s disease, familial adenomatous polyposis, and diverticular disease) [1,4].

Self-management of a stoma can involve changes to an individual’s daily routine, complications, and psychological impacts such as body image concerns and dealing with stigma [5]. A systematic review of 14 studies of people with a stoma (N=1752) found that these changes caused by stoma formation can have a negative impact on their quality of life (QOL) [6]. Previous research using latent profile analysis within this population has found that groups with QOL concerns are more likely to have a hernia, be younger, and have had their stoma for a shorter amount of time compared to those in the “consistently good quality of life” group [7].

In people with a stoma, self-management interventions have been used to improve QOL, by increasing their self-efficacy (, their confidence in being able to manage their stoma [8,9]. A meta-analysis of 5 self-management interventions for people with a stoma that measured self-efficacy found a 12-point mean (scale range from 22 to 110) difference in scores between the intervention and control groups at follow-up [10]. Research has begun to suggest that individuals’ attitudes, beliefs, and affective states could vary depending on the time they are measured and the circumstances in which they are measured [11]. Within other populations, there is emerging research to suggest that both QOL and self-efficacy may not be the stable constructs we have treated them as in traditional self-management interventions. Ecological momentary assessment (EMA) studies have found that QOL and self-efficacy may fluctuate over shorter periods of time (eg, over the course of a day) and could be influenced by contextual factors such as location [12-16]. Within the current stoma research, the day-to-day fluctuations in QOL and self-efficacy are not understood, nor are contextual factors such as where a person is, what they are doing or who they are with.

EMA is a methodology in which participants are repeatedly surveyed to provide a snapshot of their thoughts and feelings at the moment that they responded [17]. This method reduces issues around recall bias and can provide data on how variables fluctuate from one point in time to another [18]. Furthermore, cross-sectional and longitudinal studies, which are the basis for much of the research within this population, are focused upon the associations of interindividual (between-person) factors whereas EMA can examine both inter and intraindividual (within-person) factors that can vary over time and setting [19]. Using EMA within this population presents an opportunity to better understand in what circumstances both QOL and self-efficacy vary and thus inform enhancements to future self-management interventions, as within-person changes have not been considered in interventions to date.

Therefore, the primary aims of this study were to assess whether quality of life and self-efficacy fluctuate over time and what variability is attributable to within- and between-person variances. Furthermore, we assessed whether contextual factors (activity, company, and location) and time of day were associated with QOL and self-efficacy over a week.

## Methods

### Design and Participants

This study involved gathering intensive longitudinal data across 7 days. Participants were recruited via social media (X [formerly Twitter] and Facebook), as well as emails sent to members of relevant charities and support groups and adverts placed in newsletters. Participants were eligible to take part if they had a bowel stoma (colostomy or ileostomy), were over the age of 18 years, and lived in the United Kingdom. Participants were required to have a smartphone, to which they were willing to download a mobile app and have access to their smartphone at various points throughout the day. This study has been reported in line with an adapted Statement: Guidelines for Reporting Observational Studies (STROBE) Checklist for Reporting EMA Studies [20].

The sample size for the current study was determined based upon reviews of previous research using EMA, which suggests that no more than 100 participants are required [21], with 60 previous studies having an average sample size of 63 participants (median 42 participants) [22]. A further review of simulation studies for EMA studies determined that the number of data points should be greater than 900-1000 [23]. For this study, there were 21 possible data points per participant; with an average EMA protocol compliance rate of 80%, we would expect roughly a completion of 17 data points. Therefore, we required at least 59 participants to have more than 1000 data points.

### Ethical Considerations

Ethical approval for this study was provided by the University of Leeds Medicine and Health Ethics Committee (reference number MREC 20‐043). Before any data collection, participants were emailed a participant information sheet, which detailed how their data would be collected and stored on University of Leeds servers in password protected folders. All participants provided informed consent before participation. While the study was ongoing, their data were pseudonymized by a Study ID number with the key stored separately to the data. Upon completion of the study, the Study ID number was deleted to anonymize the data along with all personal information. Participants completing 80% (17/21) or more of the timed surveys were sent a US $15 Amazon gift voucher as compensation, which they were informed of during enrollment to the study.

### Procedures

Before ethics being obtained, the components of this study were discussed with a steering group comprised of relevant patients, nurses, and charity and industry representatives. These individuals provided feedback on the timing of the surveys, the number of surveys triggered, as well as the wording of the measures to ensure clarity for participants.

Recruitment for this study began as the United Kingdom was emerging from COVID-19 lockdowns starting on April 19, 2021 and concluded on March 5, 2022. For this study, the app, Avicenna Research (formerly Ethica Data) [24], was used to gather data throughout the 7-day period. This app was selected due to its compliance with data protection regulations; its use in previous research studies, which demonstrates its feasibility for supporting research studies; and its suitability for hosting EMA studies. It also had the ability to function remotely with no face-to-face interaction between participants and the researchers required for its operation.

At baseline, participants were sent a link to a survey to complete demographic and clinical characteristic measures. After this was completed, they were then sent step-by-step instructions on how to download and register for the Avicenna Research app (formerly Ethica Data app) on their smartphone. Surveys were programmed to be sent to participants at 9 AM, 1 PM, and 7 PM to provide a view of participants’ quality of life over the course of the day. These surveys started triggering the day after participants had registered for the study. Participants had a 2-hour window to complete the surveys, to allow flexibility around their schedules. Participants were encouraged to report any issues they had with the app to the research team promptly so that these could be resolved.

### Measures

The full baseline and timed surveys can be accessed in MultimediaAppendices 1  2 .

#### Demographic and Clinical Characteristics

Participants were asked for their age in years (as a continuous variable), their gender identity (male, female, or other), and the type of stoma participants had (ileostomy, colostomy, or do not know). Participants were also asked the reason for the formation of their stoma (“Cancer,” “Crohn’s Disease,” “Ulcerative Colitis,” “Diverticulitis” and “Other”). Participants could specify in the “Other” category the reason for their stoma formation. The length of time a participant had their stoma was provided in either months or years; for this study, all responses were converted to months. Participants were asked whether they had any long-standing health conditions (angina, high blood pressure, liver disease etc); they could also select “other” and specify the condition. All selected conditions were summed, and a number of comorbidities variables were derived.

#### Context

Survey responses were scheduled to trigger at 3 points during the day, to assess whether the time of day in which the responses were answered accounted for the variance. A “Time of Day” variable was created with “Morning,” “Afternoon,” and “Evening” as the 3 levels and “Morning” as the reference. Each time participants answered the timed surveys, they were initially asked 3 questions to gather information on the context in which they were answering the survey. These questions were “What is your current location?,” “What are you doing?,” and “Who are you with?”. Each of the questions had multiple response options with the additional option of providing their response by selecting “Other.”

#### Self-Efficacy

Self-efficacy was assessed with a single measure on a scale from 0 to 100 (ie, At this moment right now, how confident do you feel that you can do the different tasks and activities needed to manage your stoma right now? with 0 indicating not at all confident and 100 indicating totally confident). This measure was adapted from a previous ecological momentary assessment study in another population to make it relevant for people with a stoma [25].

#### QOL

The QOL was assessed with a single measure on a scale from 0 to 100 (ie, We would like to know your overall satisfaction with your life in general right now. This scale is numbered from 0 to 100 where 100 means totally satisfied and 0 means totally unsatisfied). This was taken from the Stoma QOL Scale [26], with this single measure identified as being able to detect changes in QOL over shorter periods.

### Statistical Analyses

All statistical analysis was conducted in SPSS v28.0, IBM. Descriptive statistics were run on the demographic and clinical characteristics. For gender, no participant selected “other”; therefore this option was not included. Any participants who selected “don’t know” for type of stoma were changed to missing. For the reason for stoma formation, “Crohn’s Disease” and “Ulcerative Colitis” were combined into an “Inflammatory Bowel Disease” category, and due to the low numbers of people reporting “Diverticulitis,” this was combined with the “Other” category. For each of the context questions, variables were dichotomized based upon the distribution of the data and whether they would provide a meaningful interpretation. This included location (“At home” or “Not at home”), activity (“Relaxing” or “An activity that required more cognitive or physical exertion”), and company (“Alone” or “With someone else”).

Multilevel models were conducted due to the clustering of responses within individuals. The models are able to account for both the within- and between-person variation. Initial null (no predictor) multilevel models were run to determine the amount of within- and between-person variance for both QOL and self-efficacy.

Two further multilevel models were conducted with self-efficacy and QOL as the dependent variables. Contextual variables (activity, location, company, and time of day) were included as fixed effects in the model. Important covariates, based upon previous research, were included within the model: age, time with stoma, and number of comorbidities [6,7]. Intraindividual variables, such as self-efficacy scores, location, activity, and company were person-mean centered to test for within-person associations, and then, the person-mean was centered by the grand mean to test for the between-person associations. The QOL was not included within the self-efficacy model as the relationship between them is not bidirectional. Within-person variables of location, activity, and company and within-person self-efficacy, for the QOL model, as well as time of day were included as random effects to test whether these effects varied across people. Statistical significance was assessed at 0.05. Analyses were conducted with full information maximum likelihood estimations to account for missing data.

## Results

### Descriptive Statistics

**Figure 1.**
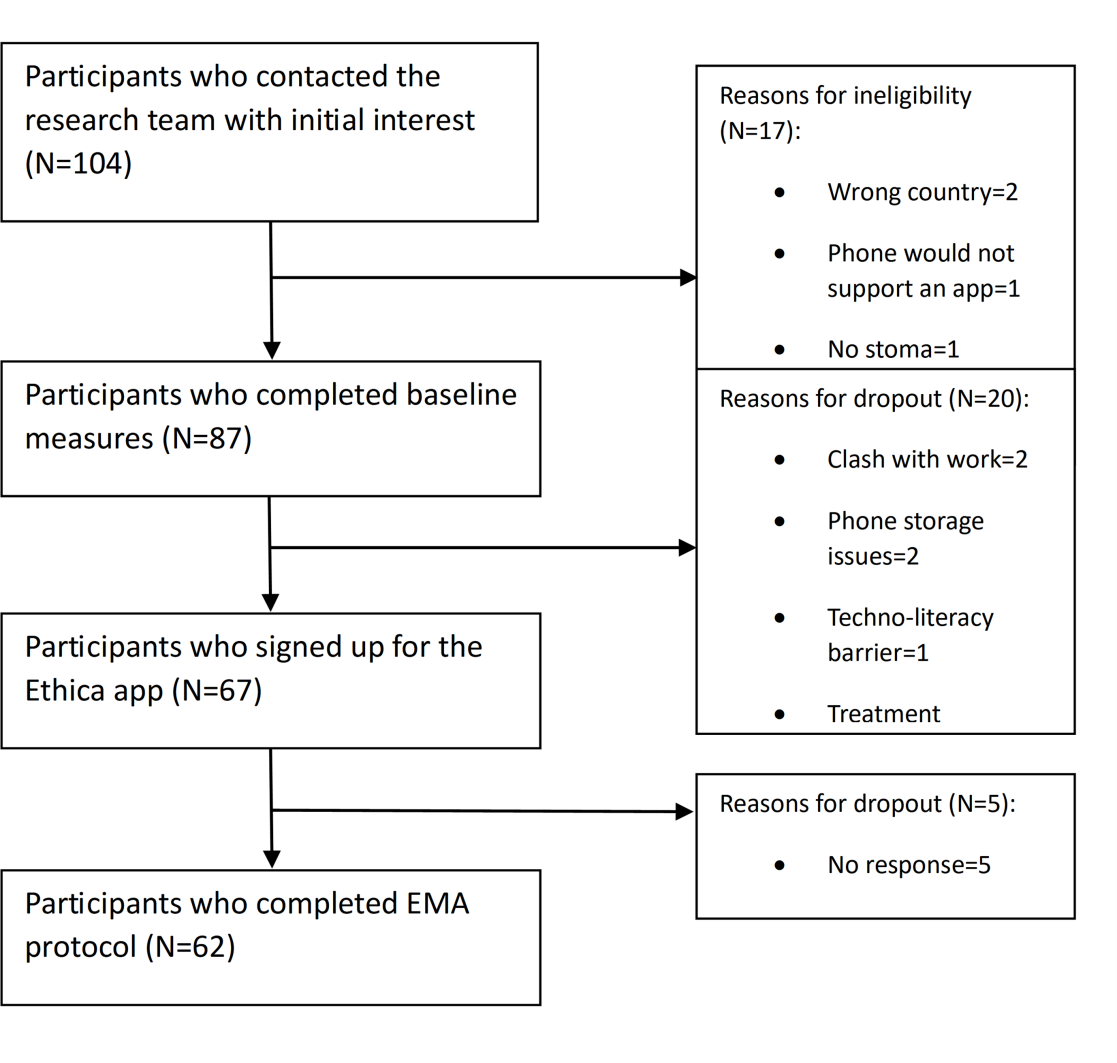
Flowchart showing the inclusion and exclusion of participants in the study. EMA: ecological momentary assessment.

A total of 62 people completed the EMA protocol, and Figure 1 outlines the flow of the participants through each stage of the study. From initial contact with the research team to completion of baseline measures, 83.7% (87/104) were eligible or remained interested in taking part in the study. From their initial interest to the completion of the EMA protocol, this dropped to 59.6% (62/104). However, from completion of baseline to completion of the EMA protocol, 71.3% (62/87) of participants remained. From a total of 1302 possible data points (62 participants × 21 time points), participants completed 1057 (81.2%), which is an average of just over 17 data points completed per person. This was almost equal across the time of day, with a range of 81‐83 missing data points for the morning, afternoon, and evening.

Table 1 provides an overview of the demographic and clinical characteristics of the sample. The sample of 62 people with a stoma consisted of mostly female participants (43/62, 69.4%), those with IBD as the reason for their stoma formation (33/62, 53.2%), and those with an ileostomy (48/62, 77.4%). The mean (SD) age of participants was 51.4 (13.9) years, with a range from 19 to 77 years, and participants had their stoma for on average just under 10 years, with a range from 3 months to 61 years. A majority of participants had at least 1 comorbid condition (36/62, 58.1%), with the most common reported conditions being asthma (11/62, 17.74%), high blood pressure (10/62, 16. 12%), and back problems (7/62, 11.29%).

**Table 1.** Descriptive statistics for the participants.

Demographic and clinical characteristics (N=62)	Values
Gender, n (%)	
Female	43 (69.4)
Male	19 (30.6)
Age, mean (SD)	51.4 (13.9)
Stoma, n (%)	
Ileostomy	48 (77.4)
Colostomy	14 (22.6)
Reason for stoma formation, n (%)	
IBD^a^	33 (53.2)
Cancer	20 (32.3)
Other	9 (14.5)
Time with stoma, (months) mean (SD)	100.4 (146.2)
Number of comorbidities, n (%)	
None	26 (41.9)
1	15 (24.2)
2 or more	21 (33.9)

a IBD: inflammatory bowel disease.

### Null Multilevel Models

No predictor models for QOL and self-efficacy indicated that the intraclass correlations for these were 0.64 and 0.65, respectively, indicating a moderate amount of stability over time. Therefore, there is still around 35% of within-person variability, which suggests there are intraindividual fluctuations over time.

### QOL and Self-Efficacy Multilevel Models

Table 2 shows the results of the multilevel models for self-efficacy and QOL. For the self-efficacy model, only the fixed effect of within-person location was statistically significant; it suggests that when individuals are outside the home, they tend to report lower self-efficacy scores (*β*=−3.7, 95% CI −6.3 to −1.2). The random effects of location suggest that associations vary significantly across persons (*β*=63.4, 95% CI 37.5 to 107.0).

**Table 2.** Multilevel model estimates for contextual effects on the quality of life and self-efficacy scores.

Variables	Self-efficacy^a^			Quality of life		
Fixed effects	Standardized *β* coefficient	95% CI	*P* value	Standardized *β* coefficient	95% CI	*P* value
Intercept	88.6	75.7 to 101.5	<.001	64.2	52.5 to 75.8	<.001
Within-person activity	−0.3	−1.4 to 0.8	.6	−1.0	−2.4 to 0.4	.2
Between-person activity	−9.7	−29.6 to 10.2	.3	16.8	−1.4 to 35.0	.1
Within-person company	−0.6	−2.5 to 1.2	.5	−1.1	−2.8 to 0.5	.2
Between-person company	0.5	−12.4 to 13.4	.9	−1.4	−13.1 to 10.2	.8
Within-person location	−3.7	*−*6.3 to −1.2	.005	2.3	−0.1 to 4.6	.1
Between-person location	4.0	−18.8 to 26.8	.7	14.4	−6.2 to 35.0	.2
Time of day (reference: morning)						
Afternoon	−0.5	−1.5 to 0.6	.4	2.1	0.7 to 3.4	.003
Evening	0.8	−0.3 to 1.8	.2	3.8	2.2 to 5.3	<.001
Within-person self-efficacy	—^b^	—	—	0.4	0.3 to 0.6	<.001
Between-person self-efficacy	—	—	—	0.7	0.4 to 0.9	<.001
Age (years)	0.03	−0.2 to 0.3	.8	0.4	0.2 to 0.6	.001
Time with stoma (months)	0.002	−0.2 to 0.3	.8	−0.03	−0.05 to −0.01	.003
Number of comorbidities	−0.1	−2.5 to 2.3	1.0	−1.7	−3.9 to 0.4	.1
Random effects (variances)						
Residual	53.1	47.1 to 59.8	<.001	83.4	73.6 to 94.5	<.001
ρ	0.3	0.2 to 0.4	<.001	0.4	0.3 to 0.5	<.001
Intercept	130.2	88.1 to 192.6	<.001	98.0	63.8 to 150.4	<.001
Within-person activity	—	—	—	3.9	0.3 to 45.8	.4
Within-person company	18.4	5.1 to 66.1	.1	4.9	0.4 to 60.3	.4
Within-person location	63.4	37.5 to 107.0	<.001	36.1	15.4 to 84.4	.02
Time of day (reference: morning)						
Afternoon	—	—	—	8.5	2.8 to 25.6	.1
Evening	—	—	—	14.2	6.0 to 33.8	.02
Within-person self-efficacy	—	—	—	0.1	0.04 to 0.2	.02

a Within- and between-person self-efficacy were not included in the self-efficacy model, and the random effect of time of day and within-person activity were not included in the self-efficacy model due to convergence issues; significant random effects indicate substantial variation in the scores across individuals.

b not applicable.

For the QOL model, there were statistically significant associations with the time of day the assessment was reported, with QOL scores increasing throughout the day (afternoon: *β*=2.1, 95% CI 0.7-3.4, evening: *β*=3.8, 95% CI 2.2-5.3). Furthermore, people with a stoma who had a higher average score of self-efficacy over the week also reported statistically significant higher QOL scores at each assessment point (*β*=.7, 95% CI 0.4-0.9). Similarly, for the within-person scores of self-efficacy, those individuals who reported a higher self-efficacy score at a given time point also tended to report higher QOL scores (*β*=.4, 95% CI 0.3-0.6). The random effect of within-person self-efficacy indicates that despite finding a fixed relationship between self-efficacy and QOL, patterns differed significantly between individuals (*β*=.1, 95% CI 0.04-0.2).

## Discussion

### Principal Findings

This study is the first exploration of the within- and between-person variances enabled by EMA methodology in people with a stoma. We were able to identify that both QOL and self-efficacy are not stable constructs for this population and that their scores fluctuated within individuals over the measurement period. For self-efficacy, individuals felt more confident in being able to manage their stoma when they are within the home environment, although there is also substantial variability in those scores. For QOL, individuals were more likely to report higher scores as the day progressed with scores on average 2 points higher in the afternoon and 4 points higher in the evening. Also, both within- and between-person self-efficacy scores were associated with QOL, indicating that higher confidence in being able to manage their stoma is associated with greater QOL scores at any given time.

This study found that QOL scores over the week were associated with contextual factors. The time of day that participants were submitting reports was associated with QOL, with lower scores in the morning and scores improving as the day went on. Previous research among people with a stoma has suggested that sleep disturbances could impact their QOL due to having to wake, possibly multiple times, during the night to change their stoma bag [27]. This could have had an impact on their QOL scores in the morning, which have then improved in the afternoon and evening. Future research should consider including a sleep quality questionnaire in an EMA study to see whether this association with QOL is influenced by time of day. This study also found that the length of time with a stoma was negatively associated with QOL, which may suggest that the additional time spent managing their stoma in the morning may impact upon their QOL while they are adjusting to having their stoma and their new routine [28].

Furthermore, a previous review of EMA studies in healthy subjects found greater well-being when participants were in a natural environment and lower scores when participants were at work [29]. While the present study did not find a statistically significant association between location and QOL, this may be due to the difference in environments compared (eg, at home vs outside of the home). Further EMA studies in people with a stoma are needed to explore how the location where an individual responds is associated with QOL in more detail by comparing more environments.

This study also found an association between both within- and between-person self-efficacy and QOL. This suggests that people who are on average more confident in managing their stoma and also individuals with greater confidence within the moment are more likely to report higher QOL scores. These findings are consistent with previous research in this population that found a positive correlation between self-efficacy and QOL scores [30]. However, the findings from this present study also suggest that not only do we need to increase self-efficacy among people with a stoma but that we also need to ensure that people with a stoma are confident in a variety of different contexts.

This is further reinforced in the self-efficacy model from this study in which we found that the location people were reporting from was associated with self-efficacy. For example, when people were at home, they reported greater confidence in being able to manage their stoma than when they were outside of the home. This may be because people with a stoma are in an environment in which they are familiar and have access to all the resources and facilities they need to manage their stoma. Previous research has also highlighted that travel is a considerable source of worry for people with a stoma [6]. Just-in-time adaptive interventions (JITAIs) are an approach to interventions whereby advice and support is offered in the moment based upon responses to certain measures or based upon contextual factors (eg location) [31,32]. Therefore, future research should consider using JITAIs to look at improving self-efficacy for individuals when they are outside the house and offering practical solutions to help them feel confident in managing their stoma in environments that are not familiar to them. Furthermore, random effects findings suggest there was substantial variability for the location variable in both the self-efficacy and QOL models as well as self-efficacy, for the QOL model, suggesting that participants do not all experience similar associations; therefore, JITAIs could provide more tailored and adaptive interventions for participants.

### Strengths

There were strengths in the design of the study and execution of the EMA procedure. The data suggested that compliance with the surveys was high, with only 19% (1057/1302) missing data points. Systematic reviews of EMA studies have found mean compliance rates ranging from 71% to 86%; therefore, the present study had compliance rates on the higher side of the average [22,29,33,34]. Furthermore, the design of the study facilitated data collection from participants in the moment, potentially reducing errors arising from retrospective recall [18].

### Limitations

However, there were limitations of this study. First, given the timing of when this study was conducted, it is possible that associations could be linked to the gradual lifting of COVID-19 lockdown restrictions, which began in early 2021 and continued into mid-2021 [35]. This study also required participants to have a smartphone and some digital-literacy to download and install apps, which may have made some groups such as older individuals ineligible to take part. However, smartphone use and digital-literacy are growing in older populations [36]. Despite this, as our recruitment techniques were primarily facilitated through the use of the internet, we may have recruited a younger sample, which may not be representative of the wider stoma population [37]. Therefore, further EMA studies are needed in the future with a larger, more diverse sample of people with a stoma. Finally, due to wanting to ensure that additional burdens were not placed upon participants, single measures of QOL and self-efficacy were used in this study. These measures provide a general overview of the constructs but would not provide granular detail of differences in the dimensions of these measures that have been observed in previous research [7]. Furthermore, these measures did not have defined clinically significant differences; therefore, further EMA studies are needed with measures that have defined clinical significant differences to understand whether the statistically significant results have clinically relevant findings [38]. The present study took a cautious approach to the inclusion of covariates in the model based upon previous research; however, future research should also consider including further covariates, such as the type of stoma, to explore whether these are associated with self-efficacy and QOL in the moment.

### Conclusion

This study is the first investigation into the variability of QOL and self-efficacy that identified fluctuating scores over a week in a population of individuals with a stoma. The findings suggest that future research could explore the effect of bolstering self-efficacy for self-management in people with a stoma at different times throughout the day to observe whether this can enhance their QOL. Differential associations between self-efficacy and QOL in certain contexts suggests tailored interventions may be needed, with some individuals likely to particularly benefit from interventions designed to boost self-efficacy, and support self-management, outside of the home environment. Additional studies using EMA methodology in people with a stoma are needed to further explore the associations found in larger, more representative samples, and to test the potential for JITAIs to improve self-management and in turn, QOL, for this population.

## Supplementary material

10.2196/57427 Multimedia Appendix 1 Baseline questionnaire.

10.2196/57427 Multimedia Appendix 2 Repeated EMA questionnaire.

## Abbreviations

EMA: ecological momentary assessment
IBD: inflammatory bowel disease
JITAI: just-in-time adaptive intervention
QOL: quality of life
STROBE: Statement: Guidelines for Reporting Observational Studies
