# Supplementary material for: Ecological Momentary Assessment of the Quality of Life and Self-Efficacy Among People With a Stoma: Longitudinal Study
Source: J Med Internet Res. 2025 Jul 2;27:e57427. doi: 10.2196/57427 (PMC12239683; doi:10.2196/57427)
Supplement: Multimedia Appendix 1 [file jmir-v27-e57427-s001.docx]

**Multimedia Appendix 1**

**Baseline questionnaire**

You are being invited to participate in a research study titled 'Understanding day-to-day well-being and self-management practices in people with a stoma'. This study is being done by William Goodman from the University of Leeds.

The purpose of this research study is to understand how individuals manage their stomas day-to-day, and how their well-being and confidence changes throughout the day. This survey will take you approximately 8 minutes to complete. Your participation in this study is entirely voluntary and you can withdraw at any time without providing a reason.

We believe there are no known risks associated with this research study; however, as with any online related activity the risk of a breach is always possible. To the best of our ability your participation in this study will remain confidential, and only anonymised data will be published. We will minimise any risks by keeping your personal information entirely confidential in a password protected folder on University of Leeds servers. This will be deleted once the research is completed. Further information is available via the University of Leeds [Privacy Notice](https://dataprotection.leeds.ac.uk/wp-content/uploads/sites/48/2019/02/Research-Privacy-Notice.pdf). For further information on this study please refer to the Information Sheet emailed to you.

What is your study ID?

What type of stoma do you have?

Ileostomy

Colostomy

Don’t know

How long have you been living with a stoma?

Years:

Months:

What was the reason for the formation of your stoma?

Cancer

Crohn’s disease

Ulcerative colitis

Diverticulitis

Physical trauma

Other please specify:

What is your gender?

Male

Female

Other

Prefer not to say

Does your gender identity match your sex as registered at birth?

Yes

No

Prefer not to say

What is your age? Please specify in years

What is your marital status?

Married/living with partner

Single

Divorced

Widowed

Other

You only need to answer one of the two following questions.

What is your height in cm?

What is your height in ft/in?

You only need to answer one of the two following questions.

What is your weight in kg?

What is your weight in st/lbs?

What is your smoking status?

Current smoker

Ex-smoker

Never smoked

How many abdominal surgeries have you had (including your stoma surgery)?

Do you currently have a parastomal hernia or a bulge around your stoma, making one side stick out more?

Yes

No

Don’t know

Have you had a parastomal hernia or a bulge in the past?

Yes

No

Don’t know

Do you have a long-standing health condition?

Yes

No

Which, if any, of the following conditions do you have?

Alzheimer’s disease or dementia

Angina

Arthritis

Asthma or other chronic chest problem

Blindness or visual impairment

Deafness or hearing impairment

Diabetes

Epilepsy

Heart condition

High blood pressure

Kidney disease

Learning difficulty

Liver disease

Long term back problems

Long standing mental health problem

Long standing neurological problem

Do not have a long standing condition

Another long-standing condition (please specify)

Thank you for completing the questions. Someone will be in touch with the next steps.
