# Supplementary material for: Ecological Momentary Assessment of the Quality of Life and Self-Efficacy Among People With a Stoma: Longitudinal Study
Source: J Med Internet Res. 2025 Jul 2;27:e57427. doi: 10.2196/57427 (PMC12239683; doi:10.2196/57427)
Supplement: Multimedia Appendix 2 [file jmir-v27-e57427-s002.docx]

**Multimedia Appendix 2**

**Repeated EMA questionnaire**

Are you happy to continue taking part in this research?

Yes

No

What is your current location?

At home (choose if working from home)

At work

Travelling

Out shopping

At a friend or family member’s house

Out for recreation

In a healthcare setting (GP surgery/Hospital)

Other please specify

What are you doing?

Working

Commuting

Exercising

Shopping

Relaxing

Socialising

Housework/Chores

Other please specify

Who are you with?

Family/Partner

Friends

Colleagues

Alone

Other please specify

We would like to know your overall satisfaction with your life in general RIGHT NOW. This scale is numbered from 0 to 100 where 100 means totally satisfied and 0 means totally unsatisfied. Drag the dial on the scale to indicate how your well-being is RIGHT NOW.

At this moment RIGHT NOW how confident do you feel that you can do the different tasks and activities needed to manage your stoma right now? 0 (not at all confident) - 100 (totally confident). Drag the dial on the scale to indicate how confident you feel RIGHT NOW.

Over the past 2 hours which of the following stoma management practices have you conducted?

Changed/emptied stoma appliance

Cleaned skin around stoma

Wore a support garment (vest, underwear or belt)

Performed exercises to strengthen your core muscles

Used deodorant to reduce odour

Dietary management

Other (please specify)

Over the past 2 hours which of the following resources/facilities/support, if any, have you had to access to manage your stoma?

Toilet facilities

Ostomy supplies

Support from partner/friend

Other (please specify)

Have needed resources/facilities but could not access them

Did not need access to resources/facilities
